# Supplementary material for: Risk Assessment of Industrial Microbes Using a Terrestrial Mesocosm Platform
Source: Microb Ecol. 2023 Dec 11;87:12. doi: 10.1007/s00248-023-02321-8 (PMC10710964; doi:10.1007/s00248-023-02321-8)
Supplement: Supplementary file 1 [file 248_2023_2321_MOESM1_ESM.docx]

**Supplement:**

**B**

**A**

**Supplemental Figure 1.** CAD drawing of 3D printed pots showing sampling ports along the side A) side view and B) top view the perforated bottom that allows for root formation and self-watering from a reservoir.

**Supplemental Figure 2. Ratios of total fungal biomass to bacterial biomass** from Fig. 3 B,C by phospholipid fatty acid (PFLA) analysis by Gas Chromatography (GC) of microbial composition of soils at baseline and 30 days post amendment.

**Supplemental Figure 3. Beta-diversity of samples over time course**. Principal coordinate 1, principal coordinate 2, and days since the experiment start to visualize how these samples changed over time.

**Supplemental Figure 4. Beta-diversity as a function of treatment (color) time (shape) and strata (scale)** of soil mycobiome (top) and bacteriome (bottom) with Bray-Curtis distance matrix.
